# Effects of once- versus twice-weekly eccentric resistance training on muscular function and structure in older adults: a randomised controlled trial

**DOI:** 10.1038/s41598-024-59788-9

**Published:** 2024-04-26

**Authors:** Brett A. Baxter, Anthony W. Baross, Declan J. Ryan, Stepan Tkadlec, Anthony D. Kay

**Affiliations:** 1 Centre for Physical Activity and Life Sciences, Faculty of Art, Science and Technology, University of Northampton, Northamptonshire, NN1 5PH UK; 2 Healthy Longevity Clinic, 165 East Palmetto Park Road, Boca Raton, FL 33432 USA; 3 Healthy Longevity Clinic, Zlatniky-Hodkovice, 252 41 Prague, Czech Republic

**Keywords:** Ageing, Quality of life

## Abstract

Adherence rates to current twice-weekly strength training guidelines are poor among older adults. Eccentric-only training elicits substantial improvements in muscle function/size so the aim of this study was to compare the effects of once- versus twice-weekly eccentric training programmes on muscle function/size in older adults. Thirty-six participants (69.4 ± 6.0 yr) were randomised into non-active control, once-, or twice-weekly training groups. Lower-limb muscle power, strength, and size were assessed at baseline, mid-, and post-eccentric training. Training was performed for 12 min per session at 50% of maximum eccentric strength. Significant increases in power (13%), isometric (17–36%) and eccentric (40–50%) strength, and VL muscle thickness (9–18%) occurred in both training groups following 12 weeks. Minimal muscle soreness was induced throughout the 12 weeks and perceived exertion was consistently lower in the twice-weekly training group. One weekly submaximal eccentric resistance training session over 12 weeks elicits similar improvements in neuromuscular function compared to the currently recommended twice-weekly training dose. Given the substantial improvements in neuromuscular function and previously reported low adherence to current twice-weekly training guidelines, eccentric training may be pivotal to developing a minimal-dose strategy to counteract neuromuscular decline. The trial was registered retrospectively on 24/01/2024 with ISRCTN (trial registration number: ISRCTN68730580).

## Introduction

Older adults, often defined as individuals ≥ 60 years of age, are key contributors to society that assist with childcare^1^, volunteer within the community^2^, and also continue to contribute towards the workforce now more than ever^3^. However, muscle mass, strength, and power decline at rates of ~ 1.0, ~ 1.5, and 3.5% per annum, respectively in older adults^4,5^. As muscle weakness^6^ rather than muscle mass^7^ is more strongly associated with fall risk, functional limitation, and physical disability^8,9^, the larger losses in muscle strength and power are of greater concern to both individuals and wider society. The health care requirements associated with an increasing older population^10^ place a financial pressure upon governments. Specifically in the United Kingdom, the cost of care associated with age-related muscle weakness is £2.5 billion per annum^11^, largely attributed to informal care (the care provided to perform everyday tasks), which is associated with functional disability. Furthermore, the World Health Organisation’s recent *“*healthy ageing*”* work focuses on the preservation of functional ability^12^. Given the issues highlighted above, developing prehabilitation and rehabilitation strategies that prevent, delay, or reverse the deterioration of neuromuscular health is essential to maintain independence, improve quality of life, and ease the financial and societal stressors placed upon governments worldwide.

Resistance training is one of the most efficacious strategies to increase muscular size and strength in older adults^13^. However, older adults often have comorbidities that make it harder to tolerate physical activity, which could make it difficult to meet the Chief Medical Officer’s current recommendation in the United Kingdom of two strength training sessions per week^14^, reflected by declining adherence rates to these guidelines across the life course^15^. Key barriers reported by older adults to achieving the twice-weekly recommendation include transport, weather, lack of time or commitment, and the fear of falling whilst exercising^16,17^, thus it has been proposed by the Older Adults Expert Working Group that future research is needed to develop resistance training strategies that require one weekly session^18^.

Paschalis et al.^19^ have demonstrated that one weekly eccentric resistance training is sufficient to improve strength in younger adults, which may be attributed to greater hypertrophic adaptations elicited by eccentric resistance training compared to traditional resistance training^20,21^. Furthermore, the eccentric-specific adaptations (selective hypertrophy of type IIx fibres, sarcomerogenesis, and potential increases in type IIx composition) result in a faster phenotype^20,22^, improving muscle function and mobility in older adults^23^. Eccentric-only resistance training is also less physically demanding (lower oxygen consumption, muscle activity, and heart rate)^24,25^ with consistently lower levels of self-perceived exertion^26,27^ than traditional resistance training (i.e. cyclical repetitions of eccentric and concentric muscle actions). The lower metabolic cost and perceived exertion make it an ideal exercise to prescribe to older adults that often have impaired physical function, which results in exercise-intolerance to traditional exercise^28^. Collectively, these findings are indicative that eccentric exercise may provide a more potent stimulus to produce a greater adaptive profile compared to traditional methods. Despite researchers suggesting that eccentric-specific exercise may be key to developing a minimal-dose resistance training programme^29–31^, the influence of weekly eccentric resistance training frequency on training adaptations and adherence in older adults has not yet been examined^30^. In younger adults, training frequency of multi-joint eccentric-only resistance training does not appear to affect short term (four weeks) training adaptations when matched for training volume, indicative of a time-efficient training modality to improve muscle function^32^. Therefore, the aim of this study was to examine and compare the effects of a once- versus twice-weekly 12-week multi-joint eccentric training programme (unmatched training volume) on the muscular function and structure of older adults. It was hypothesised that (1) once- and twice-weekly eccentric training would significantly improve muscular function and structure, and (2) twice-weekly training would induce significantly greater adaptations than once-weekly training.

## Materials and methods

CONSORT reporting guidelines^33^ have been followed where possible.

### Participants

To determine the necessary sample size to ensure adequate statistical power for all variables, effect sizes (Cohen’s *d*) were calculated from previous studies^28,34^ employing similar procedures from mean ± SD changes in muscle strength and sit-to-stand test (STS) performance. A priori power analysis using G*Power (v.3.1 Düsseldorf, Germany) was conducted using strength as it consistently had a smaller effect size than STS in the literature) using the following parameters: α = 0.05, β = 0.20, *d* = 1.56. The analysis revealed a minimum sample size of eight participants per group, with 14 participants per group recruited to account for potential participant attrition and data loss. Forty-two community-dwelling older adults (Table 1) began the training programme after providing informed written consent and completing an inclusion criteria questionnaire to determine (1) ≥ 60 years of age, (2) able to independently ambulate without walking aids, (3) were recreationally active, (4) free from any illnesses and/or medication that affected the neuromuscular system or balance, and (5) not currently involved in a structured exercise programme. The study was approved by the University Research Ethics Committee and was conducted in accordance with the Declaration of Helsinki. The training and data collection took place at the Health and Performance Laboratory at University of Northampton between September 2019 to March 2021. The trial was registered retrospectively with ISRCTN (trial registration number: ISRCTN68730580). Thirty-eight participants returned to follow-up with four withdrawals (Control [CON]; *n* = 1, once-weekly [G1X]; *n* = 1, and twice-weekly [G2X]; *n* = 2) due to musculoskeletal injuries unrelated to the training. Two participants were also removed from statistical analyses as one participant in G1X had extreme values (statistical outlier [> 3 SD above mean]) for all muscular function and size metrics and one participant in G2X had a training adherence of 67% (below the 80% threshold). Statistical analyses were conducted on 36 participants (CON; *n* = 13, G1X; *n* = 12, and G2X; *n* = 11).

**Table 1:** Participants’ demographics at baseline (mean ± SD).

Group	CON (*n* = 14)	G1X (*n* = 14)	G2X (*n* = 14)
Age (y)	67.2 ± 5.4	70.5 ± 6.3	70.4 ± 5.9
Height (cm)	167.9 ± 6.6	166.7 ± 9.5	168.7 ± 9.7
Mass (kg)	81.7 ± 18.2	75.5 ± 14.2	75.0 ± 12.9
BMI (kg·m^−2^)	28.9 ± 5.6	26.7 ± 4.3	26.3 ± 3.8
Sex (m/f)	5/9	8/6	6/8
Training adherence (%)*	N/a	96.2 ± 5.5	89.9 ± 9.0

* Participants that withdrew from the study due to unrelated injury were not included in the calculation of adherence.

### Protocol overview

A parallel randomised control trial study design was implemented with participants allocated to a non-active control group (CON; *n* = 14) who maintained normal-living conditions, and once- (G1X; *n* = 14) or twice-weekly (G2X; *n* = 14) training groups using a computerised random number generator (simple random assignment) with a ratio of 1:1:1; all randomisation procedures were conducted by BAB. A familiarisation session was included two weeks prior to the initial data collection session. During week 1, baseline values were collected for all variables and the 12-week eccentric resistance training commenced, with identical data collection sessions conducted mid- (week 7) and post-training (week 13). CON partook in the study ~18 months after the training groups due to a nationwide lockdown but were randomly allocated at the beginning of the study alongside the training groups.

### Eccentric resistance training intervention

All training procedures have been detailed in line with the Consensus on Exercise Reporting Template (CERT) guidelines^35^. The intervention lasted for 12 weeks, was performed individually on a recumbent stepper ergometer (Eccentron, Baltimore Therapeutic Equipment, Hanover, MD, USA), and was supervised by the same experienced researcher at the University Health and Performance Laboratory. Maximum eccentric force was established on the isokinetic stepper ergometer at baseline to determine the target force for an intensity of 50% of maximum. As maximum eccentric force was expected to increase during the training programme, it was assessed bi-weekly (weeks 1, 3, 5, 7, 9, 11) to maintain a relative training intensity of 50% of maximum eccentric force (detailed below). If maximum eccentric force was lower than the previous bi-weekly value, the higher value was used to ensure training load did not regress. A minimum of 48 h between sessions was administered to ameliorate any exercise-induced muscle soreness.

The following training durations were conducted at 50% of maximal eccentric force but also included an additional 1-min warm-up and 1-min cooldown performed at 25% of maximum eccentric force at 18 step‧min^−1^. To minimise the potential for muscle soreness, a progressive programme was used. Participants trained at 50% of maximum eccentric force, at 18 step‧min^−1^, for 7 min (9 min total training time including a 1-min warm-up and a 1-min cooldown) in week 1 (126 repetitions per limb), 9 min in week 2 (162 repetitions per limb), and 12 min in week 3 (216 repetitions per limb), similar to those implemented by Kay et al.^27^. From weeks 4–12, participants maintained the training duration and intensity, but step frequency was increased to 24 step‧min^−1^. After each session mechanical work performed was recorded, alongside rating of perceived exertion (RPE) using the Borg CR10 scale^36^.

To perform the eccentric resistance training, the seat was adjusted so the knee could not extend > 150° (180° = full extension) and the stride position was set so that the knee did not flex to < 90° to minimise possible injury, with a handheld stop button allowing the participant to terminate the exercise at any point. To elicit an eccentric contraction the footplates on the stepper ergometer moved towards the participant in an alternating manner (i.e. as one footplate moved towards the participant the opposing footplate moved away). Participants were instructed to resist the footplate unilaterally, alternating between limbs as the footplate moved towards them, resulting in alternating unilateral eccentric contractions of the hip extensors, knee extensors and plantar flexors, and to relax as the footplate moved away. During the exercise, the real-time visual display of force was provided that allowed participants to stay in rhythm with the stepper and match force application with reference to a pre-set target intensity (acceptable range = 40–60% maximum eccentric force); verbal encouragement was provided throughout to stay within rhythm and target intensity range.

Exercise adherence was calculated as a percentage of session attendance during the 12-week intervention. No incentivisation was implemented to enable the effect of weekly frequency on adherence to be examined, however training days and times were scheduled around the participants’ availability. No adverse events occurred throughout the training programme.

### Sessional metrics

Mechanical work was extracted from the stepper ergometer following each training session, calculated as the eccentric force multiplied by the distance of each repetition; within G2X the sum of mechanical work from the two weekly training sessions was used for subsequent analysis. RPE was recorded following each training session using the Borg CR10 scale^36^; within G2X the mean of the two weekly training sessions was used for subsequent analysis. Muscle soreness was self-reported 24 and 48 h after each training session by performing a squat movement to approximately 90° of knee flexion at the beginning of each day and rating their muscle soreness on an 11-point visual analogue scale (0 = “no pain”; 10 = “worst pain possible”). Again, within G2X the mean of the muscle soreness scores were used for subsequent analysis. Mechanical work and RPE were analysed and reported bi-weekly to align with the bi-weekly maximum eccentric strength assessments.

### Outcome measures

#### Vastus lateralis muscle structure

In vivo muscle structure of the *vastus lateralis* (VL) was examined using real-time two-dimensional B-mode ultrasonography, which was a secondary outcome measure. For imaging of the VL, participants were seated with a knee angle of 90° (180° = full extension) and the probe placed on the mid-point between the greater trochanter and lateral femoral condyle and positioned longitudinally and parallel to the direction of the muscle fibres. Once the deep and superficial VL aponeuroses were clearly visible an image was captured; the probe was then removed and re-applied to capture a second image. Images were exported and analysed using digitising software (ImageJ 1.46r, National Institutes of Health, Bethesda, MD, USA). VL muscle thickness was measured as the distance between the deep and superficial aponeuroses with three measurements taken from both images (six measurements) and the mean used for subsequent analysis. Fascicle angle was defined as the angle between the muscle fascicle and the deep aponeurosis; three fascicles were measured on both images and the mean value was used for further analysis. As the full length of the VL fascicles did not fit on the sonograph, fascicle length was estimated by trigonometry.

#### Lower-limb power

Lower-limb muscular power was a primary outcome variable and was assessed via a 10-repetition sit-to-stand (STS) test with the time to complete 10 repetitions recorded using a stopwatch to the nearest 0.01 s (the trial ended when the participant was fully stood up on the 10th repetition). The assessment was performed twice with a 1-min rest between trials and the fastest trial used for subsequent analysis. The height of the chair, body mass, and lower-limb length (distance from the greater trochanter to the lateral malleolus) of each participant were measured so that power could be calculated using previous methods^37^, see Eq. (1):

1 $${\text{Power}} = \left( {{\text{Body}}\;{\text{Mass}} \cdot {\text{g}} \cdot \left[ {{\text{Leg}}\;{\text{Length}} - {\text{Chair}}\;{\text{Height}}} \right] \cdot 10} \right) \cdot {\text{Time}}^{ - 1}$$

where g = acceleration due to gravity, Time = time to complete 10 STS repetitions, 10 = ten repetitions.

#### Lower-limb contractile ability

Dynamometry was used to assess rate of torque development (RTD), contractile impulse, and knee extensor torque during maximum voluntary isometric contractions (MVIC). The participants were seated on the dynamometer chair (Biodex System 3 Pro, IPRS, Suffolk, UK) with the hips flexed to 95° (180° = full extension), and the right knee flexed to 110°; i.e. the approximate angle whereby peak knee extensor strength is produced in older adults^38^. The right lateral femoral condyle was aligned with the axis of rotation on the dynamometer and shank strapped to the lever arm of the dynamometer attachment. Prior to maximum efforts, participants performed three submaximal unilateral isometric contractions at 50 and 75% of perceived maximum, with non-elastic strapping over the waist and arms folded across the shoulders to minimise extraneous movement. Immediately prior to initiating the test, participants were instructed to develop a small level of pre-tension (< 10 N·m) to reduce the amount of force dissipation into the cushioning on the lever arm^39^. Following submaximal efforts, participants performed five rapid contractions as “*fast* and hard” (with the emphasis on fast) as possible, with each contraction separated by 15 s rest. If a trial displayed signs of countermovement (visually checked for an initial reduction in torque) it was deemed invalid, and the test was repeated.

RTD (the slope of the torque-time trace [Δtorque‧Δtime^−1^]) and impulse (the area under the curve of the torque-time trace [∫torque d*t,*]) were secondary outcome measures calculated from the onset of contraction over several epochs (0–100, 0–150, 0–200, 0–250, and 0–300 ms); peak RTD (RTD_peak_) was examined using a rolling 20-ms epoch^40^. The onset of muscular contraction (0 ms) was determined manually using visual inspection of the inflexion point on the torque-time trace in a figure with a y-axis (torque) scale of ~ 1 N·m and an x-axis (time) scale of ~ 200 ms^41^ as this has demonstrated greater accuracy than automated methods^42^. RTD and impulse data were extracted from the five explosive contractions with the mean of the three most explosive trials (greatest RTD over all epochs) used for subsequent analysis^42^.

Following the rapid contractions, participants performed ramped MVICs over a 5-s epoch, which was a primary outcome measure. The MVICs were initiated from rest with participants instructed to reach maximum after ~ 3 s and continue to contract “as hard as possible” to enable a 2-s plateau and confirm that MVIC had been reached. Following a 1-min rest, participants repeated the contraction until three valid trials were collected. The highest value of isometric torque from the three maximal trials was used for subsequent analysis. Joint torque data during these trials were directed from the dynamometer to a high-level transducer (HLT100C, Biopac, CA, USA) before analogue-to-digital conversion with data sampled at 2000 Hz (MP150 Data Acquisition, Biopac, CA, USA). Data were directed to a personal computer (Elitebook, HP Inc., CA, USA) running AcqKnowledge software (v.4.4, Biopac). Subsequently, data were smoothed off-line in RStudio (v.1.0.153, RStudio, Inc., MA, USA) with a custom-written fourth-order, zero-lag Butterworth filter at 150 Hz^43^.

Eccentric lower-limb force was a primary outcome measure and was assessed on a recumbent isokinetic stepper ergometer alternating unilateral eccentric contractions. Participants performed two submaximal warm-up sets of 12 unilateral repetitions (six per limb) at 25 and then 50% of their maximal effort. Subsequently, participants performed two sets of 12 maximal efforts, resisting the foot plates as they moved towards them unilaterally with a 1-min rest between the sets; the highest value of both limbs used for subsequent analysis.

### Statistical analyses

Statistical analyses were conducted using SPSS for Windows (v.28 IBM Corp., Armonk, NY, USA). Normality of distribution was examined via Shapiro–Wilk tests with homogeneity of variance assessed via Levene’s or Mauchly’s tests. Data that failed the assumption of normal distribution were transformed (natural logarithm or square root). Data that continued to fail to meet the assumption of normal distribution were analysed using non-parametric tests (Kruskal–Wallis and Mann–Whitney U tests to examine between-group differences, alongside Friedman tests to examine within-group differences) and where significant differences between groups were identified at baseline, between-group differences were not examined at mid- or post-training.

Data that satisfied the parametric assumptions were analysed using a two-way mixed-model ANOVA (time × 3 [baseline, mid-training, and post-training], group × 3 [CON, G1X, and G2X]) to examine within- and between- effects; where sphericity was violated, correction factors were used. Where a significant interaction effect was detected, simple main effects analyses (pairwise comparisons) were conducted with Bonferroni correction. Tukey’s Honestly Significant Difference test was used for analyses of bi-weekly maximum eccentric force, mechanical work, RPE, and muscle soreness, due to the number of time points examined (7, 6, 6, and 12, respectively). Data that followed the parametric assumptions and were significantly different between groups at baseline (identified via a one-way ANOVA), were analysed via a baseline-adjusted ANCOVA. Standardised effect sizes were calculated to examine the magnitude of change, *r* (with 95% confidence intervals [CI]) was calculated for non-parametric analyses^44^, with partial eta squared (η_p_^2^) and Cohen’s *d* (with 95% CI) calculated for parametric analyses^45^. Group data are reported as mean ± SE and change data are reported as mean ± SD. Statistical significance for all tests was accepted at *P* < 0.05.

### Ethical approval

The study was approved by the University Research Ethics Committee and was conducted in accordance with the Declaration of Helsinki. All participants provided written informed consent prior to partaking in the study.

## Results

### Sessional metrics

#### Bi-weekly maximum eccentric force

**Figure 1:**
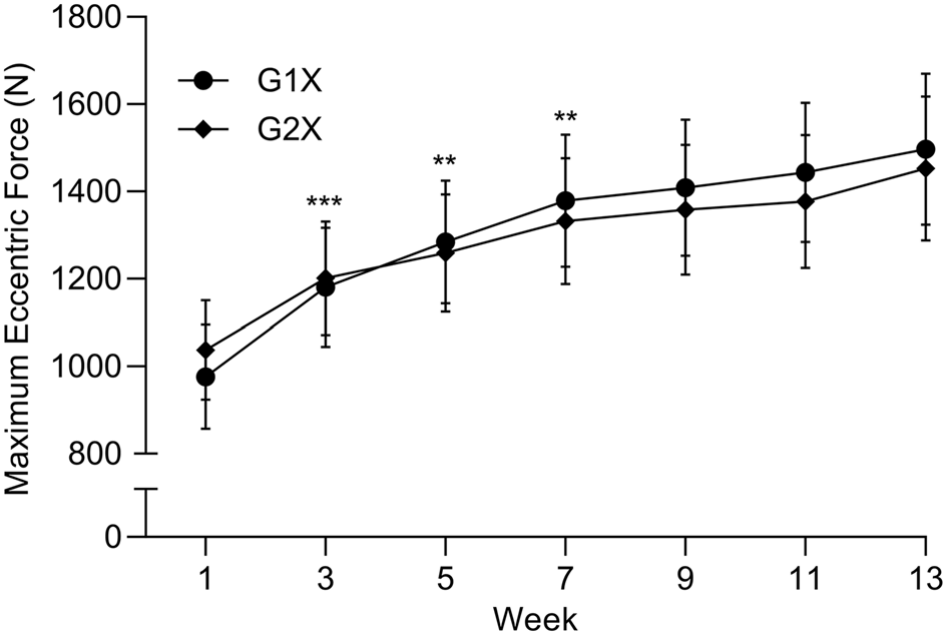
Bi-weekly maximum eccentric force (mean ± SE) throughout the 12-week training programme. Symbols (** *P* < 0.01 and *** *P* < 0.001) denote significant differences when data were collapsed across groups despite being depicted individually.

No significant interaction effect (*F*_2.35, 47.06_ = 0.373, *P* = 0.724, η_p_^2^ = 0.018) with a main effect of time (*F*_2.35, 47.06_ = 26.565, *P* < 0.001, η_p_^2^ = 0.570) but not group (*F*_1, 21_ = 0.444, *P* = 0.513, η_p_^2^ = 0.022) was detected with increases in bi-weekly maximum eccentric force in G1X and G2X occurring at similar rates over the 12-week intervention. Data collapsed across training groups revealed significant (*P* < 0.001) increases in maximum eccentric force at all time points when compared to baseline, indicating maximum eccentric force increased after only two weeks of training. Furthermore, when compared to the previous time point, significant (*P* < 0.001–0.003) increases in maximum eccentric force were evident up until week 7 indicating a slowing or plateauing of the increase in force (Fig. 1).

#### Bi-weekly mechanical work

As the study design did not standardise training volume, G2X should have completed approximately double the volume of training compared to G1X, which was confirmed by significantly (*P* < 0.001) greater mechanical work performed each week in G2X (weekly mean ± SE = 57.2 ± 7.9 kJ) than G1X (weekly mean ± SE = 26.4 ± 6.7 kJ) during the training programme. The programme was progressive in design up to week 4 of the intervention and a significant interaction effect for bi-weekly mechanical work confirmed this (*F*_2.59, 54.51_ = 8.163, *P* < 0.001, η_p_^2^ = 0.280). Significant (*P* < 0.001–0.012) increases in mechanical work (when compared to the previous time point) in both training groups were evident up until week 5, indicating that participants were consistently meeting the intensity, step rate, and duration targets of the intervention.

#### Bi-weekly perceived exertion

**Figure 2:**
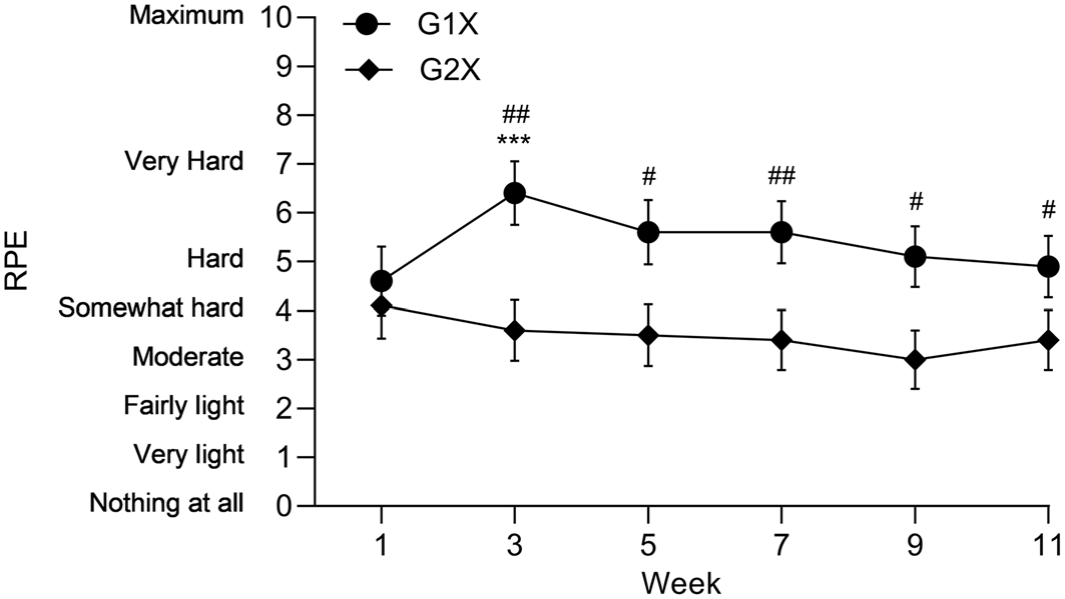
Bi-weekly RPE (mean ± SE) throughout the 12-week training programme; *** denotes a significant difference to the previous time point within G1X to *P* < 0.001, # denotes a significant between-group difference at the respective time point to *P* < 0.05, and ### denotes a significant between-group difference at the respective time point to *P* < 0.001.

Friedman tests revealed a significant effect of time for bi-weekly RPE within G1X (χ^2[10]^ = 16.603, *P* = 0.005) but not G2X (χ^2[11]^ = 2.464, *P* = 0.782). RPE significantly increased within G1X from week 1 to week 3 (4.0 ± 1.7 vs. 6.0 ± 1.7; *P* < 0.001, *r* = 0.98 [95% CI = 0.93, 1.04]) but hereafter, no significant bi-weekly differences occurred. No between-group differences in RPE were revealed during week 1 but hereafter, G1X reported significantly (*P* = 0.001–0.047) greater RPE than G2X, see Fig. 2.

#### Muscle soreness

Friedman tests revealed no significant effect of time for muscle soreness 24 h (χ^2[11]^ = 7.376–17.603, *P* > 0.05) and 48 h (χ^2[11]^ = 9.952–17.424, *P* > 0.05) post-exercise within both training groups. No between-group differences were revealed throughout the training programme for 24 h (*U* = 39.500–63.000, *P* > 0.05) and 48 h muscle soreness (*U* = 36.500–58.000, *P* > 0.05). Minimal muscle soreness was induced throughout the training programme within G1X (≤ 2/10) and G2X (< 1/10); for muscle soreness values please see publicly available data.

### Outcome measures

#### Vastus lateralis muscle structure

A significant interaction effect was revealed for VL muscle thickness (*F*_4, 64_ = 4.985, *P* = 0.001, η_p_^2^ = 0.238). Muscle thickness significantly increased within G1X from baseline to mid-training (5.8 ± 6.7% [1.1 ± 1.4 mm]; *P* = 0.037, *d* = 0.78 [95% CI = 0.73, 0.83]) and baseline to post-training (8.7 ± 8.6% [1.7 ± 1.6 mm]; *P* = 0.005, *d* = 1.05 [0.99, 1.12]). Within G2X, muscle thickness also increased from baseline to mid-training (10.2 ± 10.6% [1.8 ± 1.7 mm]; *P* < 0.001, *d* = 1.01 [0.95, 1.08]) and baseline to post-training (17.7 ± 12.3% [2.9 ± 1.9 mm]; *P* < 0.001, *d* = 1.56 [1.46, 1.66]), but unlike G1X, continued to increase from mid-training to post-training (7.0 ± 7.3% [1.1 ± 1.2 mm]; *P* = 0.023, *d* = 0.93 [0.87, 0.99]). No significant change was detected in CON. No significant difference was detected at baseline or mid-training between groups, however muscle thickness was significantly greater post-training in G1X than CON (*P* = 0.028, *d* = 0.99 [0.10, 1.88]). For group mean ± SE muscle structure metrics, see Table 2.

**Table 2:** Metrics of muscle structure over time and between groups (mean ± SE).

Measurement	Baseline	Mid-training	Post-training
Muscle thickness (mm)			
*CON*	18.0 ± 1.7	18.2 ± 1.7	18.0 ± 1.6
*G1X*	20.1 ± 1.0	21.1 ± 1.0*	21.8 ± 1.0**
*G2X*	16.9 ± 1.0	18.7 ± 1.1***	19.8 ± 1.1***†
Fascicle angle (°)			
*CON*	8.9 ± 0.8	8.2 ± 0.9	8.2 ± 0.7
*G1X*	9.9 ± 0.6	10.0 ± 0.8	11.2 ± 0.7*†##
*G2X*	8.9 ± 0.6	9.7 ± 0.8	11.0 ± 0.7***#
Fascicle length (mm)			
*CON*	118.6 ± 10.6	126.3 ± 11.9	123.0 ± 10.3
*G1X*	121.4 ± 7.6	126.1 ± 8.6	114.0 ± 5.4
*G2X*	112.6 ± 7.9	110.7 ± 9.0	103.2 ± 5.7

* Denotes a significant difference to baseline *P* < 0.05, ** denotes a significant difference to baseline *P* < 0.01, *** denotes a significant difference to baseline *P* < 0.001, † denotes a significant difference to mid-training *P* < 0.05, # denotes a significant difference to CON at the respective time point *P* < 0.05, and ## denotes a significant difference to CON at the respective time -point *P* < 0.01. CON = control group, G1X = once-weekly training group, and G2X = twice-weekly training group.

A significant interaction effect was revealed for VL fascicle angle (*F*_4, 64_ = 3.459, *P* = 0.013, η_p_^2^ = 0.178). Significant increases occurred from baseline to post-training (1.5 ± 1.4°; *P* = 0.010, *d* = 1.07 [95% CI = 1.00, 1.13]) and from mid-training to post-training (1.2 ± 1.6°; *P* = 0.041, *d* = 0.77 [0.72, 0.82]) within G1X. Within G2X, a significant increase was only detected from baseline to post-training (2.0 ± 2.2°; *P* < 0.001, *d* = 0.92 [0.86, 0.98]). No significant change was detected in CON. No significant differences were detected at baseline or mid-training between groups, however fascicle angle was significantly greater post-training in G1X (*P* = 0.008, *d* = 1.37 [0.43, 2.30]) and G2X (*P* = 0.013, *d* = 1.43 [0.51, 2.34,]) than CON.

No significant interaction (*F*_4, 64_ = 0.870, *P* = 0.487, η_p_^2^ = 0.052) or main time (*F*_2, 64_ = 2.274, *P* = 0.111, η_p_^2^ = 0.066) or group (*F*_2, 32_ = 1.285, *P* = 0.291, η_p_^2^ = 0.074) effects were revealed for VL fascicle length.

#### Lower-limb power

**Figure 3:**
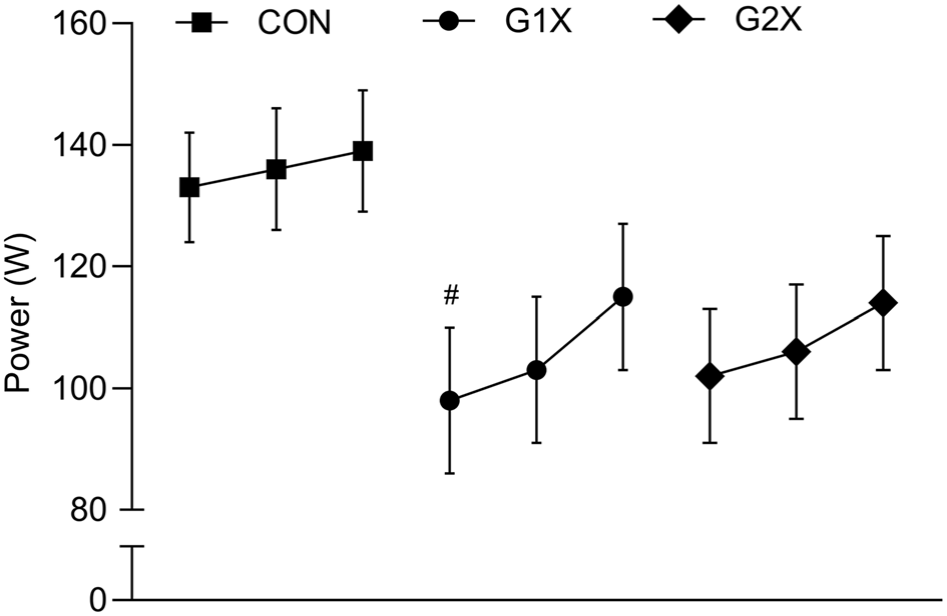
Power (mean ± SE) over time and within each training group (where lines from left to right represent baseline, mid-training, and post-training, respectively); # denotes a significant between-group difference relative to CON at the respective time point to *P* < 0.05.

A significant difference was detected at baseline (*F*_2, 33_ = 4.582, *P* = 0.018, η_p_^2^ = 0.217) with power greater in CON than G1X (133 ± 9 vs. 96 ± 7 W; *P* = 0.025, *d* = 1.30 [95% CI = 0.43, 2.16]). Consequently, a two-way baseline-adjusted ANCOVA was used that revealed no significant interaction (*F*_2, 32_ = 2.454, *P* = 0.102, η_p_^2^ = 0.133) or main time (*F*_1, 32_ = 0.014, *P* = 0.905, η_p_^2^ = 0.000) or group (*F*_1, 32_ = 0.381, *P* = 0.686, η_p_^2^ = 0.023) effects. As the baseline-adjusted ANCOVA eliminated the ability to determine early (baseline to mid-training) within-group temporal changes (potentially masking important early adaptations), a two-way ANOVA was conducted to clarify the temporal changes in the training groups only. While no interaction effect was revealed (*F*_2, 42_ = 0.586, *P* = 0.561, η_p_^2^ = 0.027), a main effect of time (*F*_2, 42_ = 9.932, *P* < 0.001, η_p_^2^ = 0.321) but not group (*F*_1, 21_ = 0.110, *P* = 0.744, η_p_^2^ = 0.005) was. Data collapsed across training groups revealed that power significantly increased from baseline to post-training (13.2 ± 13.8% [12.6 ± 13.8 W]; *P* = 0.004, *d* = 0.91 [0.85, 0.97]) and from mid-training to post-training (8.6 ± 10.0% [9.1 ± 10.1 W]; *P* = 0.007, *d* = 0.90 [0.84, 0.95]), see Fig. 3.

#### Maximum knee extensor isometric torque

**Figure 4:**
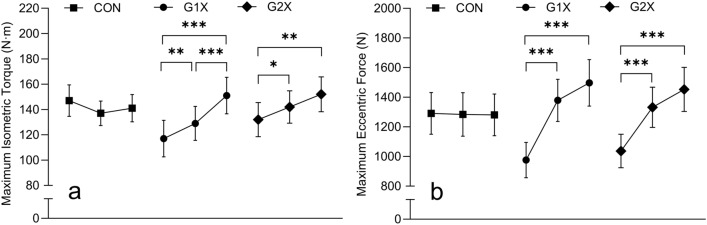
Maximum isometric torque (a) and maximum eccentric force (b) (mean ± SE) over time and within each training group (where lines from left to right represent baseline, mid-training, and post-training, respectively); * *P *< 0.05, ** *P* < 0.01, and *** *P* < 0.001.

A significant interaction effect was revealed for maximum isometric torque (*F*_*4*, 66_ = 10.789, *P* < 0.001, η_p_^2^ = 0.395). Significant increases maximum isometric torque from baseline to mid-training (12.1 ± 13.7% [13.4 ± 17.3 N·m]; *P* = 0.004, *d* = 0.81 [95% CI = 0.76, 0.86]), baseline to post-training (35.7 ± 21.9% [36.2 ± 27.2 N·m]; *P* < 0.001, *d* = 1.27 [1.19, 1.35]), and from mid-training to post-training (21.1 ± 15.0% [22.8 ± 18.3 N·m]; *P* < 0.001, *d* = 1.12 [1.05, 1.19]) occurred within G1X. Within G2X, significant increases were only detected from baseline to mid-training (10.1 ± 13.4% [10.6 ± 14.9 N·m]; *P* = 0.023, *d* = 0.71 [0.66, 0.75]) and baseline to post-training (17.1 ± 14.5% [20.3 ± 16.3 N·m]; *P* = 0.003, *d* = 1.24 [1.16, 1.32]). No significant changes were detected within CON, see Fig. 4a. No significant difference was detected at any time point between any groups.

#### Maximum lower-limb eccentric force

A significant interaction effect was revealed for maximum eccentric force (*F*_4, 64_ = 9.132, *P* < 0.001, η_p_^2^ = 0.363). Significant increases in maximum eccentric force from baseline to mid-training (34.3 ± 23.2% [338 ± 271 N]; *P* < 0.001, *d* = 1.35 [95% CI = 1.26, 1.43]) and baseline to post-training (50.2 ± 34.9% [469 ± 362 N]; *P* < 0.001, *d* = 1.38 [1.29, 1.46]) occurred within G1X. Similarly, within G2X significant increases were revealed from baseline to mid-training (30.2 ± 18.3% [294 ± 168 N]; *P* < 0.001, *d* = 1.76 [1.64, 1.87]) and baseline to post-training (40.4 ± 21.7% [415 ± 221 N]; *P* < 0.001, *d* = 1.88 [1.76, 2.00]). No significant changes were detected within CON, see Fig. 4b. No significant difference was detected at any time point between any groups.

#### Lower-limb rate of torque development

No significant interaction effect was revealed for RTD over all epochs or RTD_Peak_ (*F*_4, 64–66_ = 0.891–2.262, *P* > 0.05, η_p_^2^ = 0.053–0.124). Main effects analyses revealed significant effects of time in epochs between 0–150 and 0–300 ms (*F*_2, 64–66_ = 3.608–13.932, *P* < 0.05, η_p_^2^ = 0.101–0.296), but not RTD_0-100_ (*F*_2, 66_ = 1.093, *P* = 0.341, η_p_^2^ = 0.032). No main effects of group were revealed for any metrics of RTD (*F*_2, 32–33_ = 0.060–0.860, *P* > 0.05, η_p_^2^ = 0.002–0.031); for group mean ± SE metrics of RTD, see Table 3.

**Table 3:** Metrics of rate of torque development over time and between groups (mean ± SE).

Measurement	Baseline	Mid-training	Post-training
RTD_0-100_ (N·m·s^−1^)			
*CON*	485 ± 66	466 ± 49	480 ± 55
*G1X*	477 ± 79	471 ± 64	454 ± 70
*G2X*	442 ± 83	509 ± 67	480 ± 73
*Collapsed*	469 ± 46	481 ± 37	471 ± 41
RTD_0-150_ (N·m·s^−1^)			
*CON*	456 ± 51	457 ± 42	478 ± 40
*G1X*	444 ± 57	458 ± 51	475 ± 54
*G2X*	411 ± 60	501 ± 54	502 ± 56
*Collapsed*	438 ± 33	471 ± 30	485 ± 31*
RTD_0-200_ (N·m·s^−1^)			
*CON*	394 ± 39	395 ± 34	418 ± 31
*G1X*	357 ± 45	388 ± 42	426 ± 45
*G2X*	365 ± 47	428 ± 44	452 ± 47
*Collapsed*	373 ± 26	403 ± 24*	431 ± 26***
RTD_0-250_ (N·m·s^−1^)			
*CON*	355 ± 37	362 ± 33	378 ± 28
*G1X*	301 ± 42	337 ± 40	389 ± 43
*G2X*	332 ± 43	395 ± 42	420 ± 44
*Collapsed*	330 ± 24	364 ± 23*	395 ± 25***
RTD_0-300_ (N·m·s^−1^)			
*CON*	317 ± 36	315 ± 38	325 ± 31
*G1X*	261 ± 38	295 ± 36	351 ± 39
*G2X*	298 ± 39	358 ± 37	379 ± 41
*Collapsed*	293 ± 22	321 ± 21*	350 ± 23**
RTD_Peak_ (N·m·s^−1^)			
*CON*	742 ± 90	713 ± 71	775 ± 69
*G1X*	668 ± 110	680 ± 88	715 ± 86
*G2X*	724 ± 114	808 ± 92	798 ± 90
*Collapsed*	712 ± 63	731 ± 51	762 ± 50

“Collapsed” represents the group data collapsed at each time point; * denotes a significant difference to baseline *P* < 0.05, ** denotes a significant difference to baseline *P* < 0.01, and *** denotes a significant difference to baseline *P* < 0.001. CON = control group, G1X = once-weekly training group, G2X = twice-weekly training group, and RTD = rate of torque development.

#### Lower-limb contractile impulse

No significant interaction effect was revealed for impulse over all epochs (*F*_4, 64_ = 0.976–2.270, *P* > 0.05, η_p_^2^ = 0.056–0.124). Main effects analyses revealed significant effects of time for epochs between 0–200 ms and 0–300 ms (*F*_2, 64_ = 4.026–12.062, *P* < 0.05, η_p_^2^ = 0.109–0.268), but not for 0–100 and 0–150 (*F*_2, 66_ = 0.839–1.496, *P* > 0.05, η_p_^2^ = 0.025–0.083). No main effects of group were revealed for any metrics of impulse (*F*_2, 32_ = 0.060–0.410, *P* > 0.05, η_p_^2^ = 0.004–0.024); for group mean ± SE metrics of contractile impulse, see Table 4.

**Table 4:** Metrics of contractile impulse over time and between groups (mean ± SE).

Measurement	Baseline	Mid-training	Post-training
Impulse_0-100_ (N·m·s)			
*CON*	2.56 ± 0.34	2.42 ± 0.25	2.49 ± 0.28
*G1X*	2.49 ± 0.41	2.44 ± 0.33	2.34 ± 0.36
*G2X*	2.27 ± 0.42	2.60 ± 0.34	2.42 ± 0.37
*Collapsed*	2.45 ± 0.23	2.48 ± 0.19	2.42 ± 0.21
Impulse_0-150_ (N·m·s)			
*CON*	5.33 ± 0.57	5.26 ± 0.47	5.51 ± 0.45
*G1X*	5.14 ± 0.65	5.27 ± 0.59	5.43 ± 0.61
*G2X*	4.70 ± 0.68	5.69 ± 0.61	5.67 ± 0.64
*Collapsed*	5.07 ± 0.38	5.40 ± 0.34	5.53 ± 0.35
Impulse_0-200_ (N·m·s)			
*CON*	8.14 ± 0.79	8.06 ± 0.68	8.53 ± 0.63
*G1X*	7.34 ± 0.90	7.93 ± 0.85	8.65 ± 0.91
*G2X*	7.39 ± 0.94	8.64 ± 0.89	9.06 ± 0.95
*Collapsed*	7.64 ± 0.52	8.19 ± 0.49	8.73 ± 0.53**
Impulse_0-250_ (N·m·s)			
*CON*	11.40 ± 1.16	11.51 ± 1.03	12.02 ± 0.88
*G1X*	9.65 ± 1.30	10.72 ± 1.26	12.31 ± 1.34
*G2X*	10.47 ± 1.35	12.43 ± 1.31	13.14 ± 1.40
*Collapsed*	10.53 ± 0.75	11.53 ± 0.73*	12.46 ± 0.77***
Impulse_0-300_ (N·m·s)			
*CON*	14.62 ± 1.60	14.40 ± 1.70	14.86 ± 1.39
*G1X*	12.04 ± 1.69	13.49 ± 1.62	15.97 ± 1.77
*G2X*	13.54 ± 1.77	16.20 ± 1.69	17.05 ± 1.85
*Collapsed*	13.43 ± 0.98	14.65 ± 0.95*	15.90 ± 1.02**

“Collapsed” represents the group data collapsed at each time point; ** denotes a significant difference to baseline *P* < 0.01, and *** denotes a significant difference to baseline *P* < 0.001. CON = control group, G1X = once-weekly training group, and G2X = twice-weekly training group.

## Discussion

In agreement with the first hypothesis the main finding of the current study was that both once- and twice-weekly eccentric resistance training improved neuromuscular function. The ability of eccentric training to improve muscular strength, size, and power with once-weekly training is consistent with findings by Paschalis et al.^19^ who reported favourable strength and metabolic adaptations after one weekly eccentric resistance training session performed for eight weeks in young adults. Therefore, the current study’s finding that once-weekly eccentric resistance training resulted in comparable improvements in neuromuscular function to twice-weekly training, despite a substantially smaller mean weekly workload (26 vs. 57 kJ), demonstrates that once-weekly eccentric resistance training frequency could be a promising recommendation for older adults’ physical activity guidelines. The eccentric resistance training performed in the present study was also seated, which eliminates the risk (and fear) of falling, allowing the exercise to potentially be performed by those with functional limitations (such as poor mobility) and psychological barriers (fear of falling). Furthermore, the programme was accompanied by minimal subjective muscle soreness and “moderate” to “hard” RPE. Thus, the findings of the present study show promise for the use of eccentric resistance training programmes when a once-weekly training frequency needs to be prescribed, with further studies needed to explore the use of different eccentric exercise equipment and the effectiveness of eccentric exercise training programmes implemented into primary care settings.

### Neuromuscular function

Eccentric resistance training performed once- or twice-weekly for 12 weeks increased lower-limb muscular power (13%; *d* = 0.91) in the current study, which is more than the previously established minimum for clinically meaningful differences (9–10%) in mobility-impaired older adults^46^. The increases in power may be attributed to a shift towards a faster phenotype^20,22^, supported by the increases in explosive capacity (discussed below) and particularly, an enhanced ability to utilise the stretch–shortening cycle given the cyclic nature of the STS test^20^. As power declines at a greater rate than muscle size or strength^5,47^ and is associated with physical and cognitive impairment^48^, functional ability^8^, and the risk of falling^48^, the ability to reverse the decline in power is pivotal. However, a key observation was that power adaptations took 12 weeks to occur with no significant change evident at the mid-training point after six weeks, which should influence exercise prescription guidelines for both duration of eccentric resistance training programmes and when to employ a potentially lower volume maintenance dosage. Hence, multi-joint eccentric resistance training performed for 12 min per week can elicit improvements in muscular power, however these adaptations take longer to occur than strength adaptations and given the importance of power, healthcare practitioners should consider prescribing this training modality for a minimum of 12 weeks. A limitation of the present study was that the participants consisted of functionally independent community-dwelling older adults, although this demonstrates that the eccentric resistance training programme can delay neuromuscular decline in an otherwise healthy older population. However, those who have already been diagnosed with sarcopenia or frailty etc. may benefit more given the greater functional decline evident in these populations and potential for improvement. Therefore, further research should be conducted to evaluate the dose–response characteristics and efficacy to reverse neuromuscular decline, and tolerability/adherence of this training modality, in older clinical populations.

Unlike power, maximum isometric torque (10–12%, *d* = 0.71–0.81) and eccentric lower-limb force (30–34%, *d* = 1.35–1.76) increased after only six weeks of training in both groups, with no further increase from mid-training to post-training, although larger mean increases in isometric torque (17–36%, *d* = 1.24–1.27) and eccentric force (40–51%, *d* = 1.38–1.88) were apparent at post-training when compared to baseline in both training groups. The magnitude of change in isometric strength after 12 weeks (22–35 N‧m) is considerably greater than the minimum clinical difference associated with all-cause mortality (15 N = 7% reduction) in older adults^49^. The rapid increase in maximum eccentric force during the initial six weeks may also be due to the specificity of the intervention and assessment method given that the training procedures mimicked the protocol used to measure lower-limb maximum eccentric force, which may also explain the disparate increases in eccentric strength compared to isometric^50^. Nonetheless, the increases after 12 weeks in the present study are similar to those reported after only six weeks by Kay et al.^27^, suggesting that only six weeks are required to substantially improve eccentric strength with further significant, albeit at a slower rate, increases from seven weeks onwards. Alongside improvements in maximum strength, explosive capacity improved in all groups including CON, but effect sizes were considerably larger within the training groups in epochs ≥ 200 ms, which may not be fast enough to react to a slip, trip, or fall^51^. Given that a lot of falls occur during quiet standing^52^ or stair negotiation particularly stair descent^53^), the increases in isometric and eccentric strength are indicative of an efficacious intervention capable of reducing the risk of falling in older adults.

To maintain the 50% eccentric training intensity, maximum eccentric force was measured every two weeks, with significant increases in bi-weekly maximum eccentric force plateauing at mid-training (week 7) in both groups, which may be attributable to (or a consequence of) the lack of progression in work performed. During the initial four weeks, training volume was increased via longer training durations and faster step frequency to ease participants into the programme, which is of particular importance when performing eccentric exercise due to the potential for symptoms of muscle damage that are often experienced^54–56^. However, if managed appropriately using a progressive programme (as in the present study), disruption to neuromuscular function can be minimised in older adults^56,57^. A significant increase in RPE was reported within G1X from week 1 to week 3, with G1X also consistently reporting significantly greater RPE (12-week mean ± SE = 5.3 ± 0.7; “hard”) than G2X (3.2 ± 0.7; “moderate”), despite the lower overall weekly mechanical work (12-week mean ± SE mechanical work = 27 ± 3 vs. 57 ± 3 kJ for G1X and G2X, respectively). The lower RPE values reported in G2X are consistent with the findings of Crane et al.^32^, whereby the high frequency training group (thrice-weekly) consistently reported lower RPE than the low frequency (once-weekly) training group, although it should be noted that training volume was matched unlike the present study. Consequently, the lower RPE may be attributable to lower sessional volume in the higher frequency group or potentially the repeated bout effect, a phenomenon whereby the initial symptoms of muscle damage experienced following unaccustomed eccentric exercise are alleviated in subsequent bouts^58^. Burt et al.^59^ reported that this can result in lower RPE values, which could suggest that the greater workload performed by G2X elicited a greater protective effect than G1X. Although G1X reported RPE values that are considered “hard” on the CR10 scale^36^, the workload performed eccentrically would likely have been unachievable using traditional resistance training methods. Eccentric strength of the lower limbs is approximately 40% greater than concentric^60^, meaning that participants were training at approximately 70% of their concentric maximum throughout the programme. Despite the large workloads (relative to concentric) tolerated, minimal muscle soreness was elicited (even during the initial weeks), which align with the findings of LaStayo et al.^56^ and Baxter et al.^57^, further supporting the contention that the repeated bout effect was elicited. Therefore, when accounting for the absolute workload performed, RPE remained reasonable throughout the training programme despite the increase in absolute workload, which was accompanied by minimal muscle soreness that may also explain the high adherence rates amongst both training groups (90–96%). Collectively, the substantial improvements in neuromuscular function with minimal muscle soreness, moderate-to-hard perceived exertion, and high adherence rates are indicative of an effective training modality with important implications for exercise prescription in older adults.

### Muscle structure

Six weeks of eccentric resistance training was sufficient to increase muscle thickness in both training groups with the large improvements within G2X evident after only six weeks (~ 10%; *d* = 1.01), findings comparable to those reported by Kay et al.^27^ that performed twice-weekly sessions for six weeks (~ 10%; *d* = 1.71–2.54). However, only moderate increases were notable within G1X (~ 6%; *d* = 0.78) that appeared to plateau at mid-training, unlike G2X that continued to increase from mid-training to post-training (~ 7%; *d* = 0.93). These data are indicative that whilst one weekly training session is enough to elicit increases in muscle thickness, two weekly sessions elicit greater initial improvements, which align the findings of Morton et al.^61^ who reported that greater frequencies positively influence hypertrophy when sessional volume is not matched. Fascicle angle increased in both training groups following 12 weeks of eccentric resistance training (1.5–2.0°; *d* = 0.92–1.04); G2X plateaued at mid-training, unlike G1X who continued to increase from mid-training to post-training (1.2°; *d* = 0.81). Whilst increases in fascicle angle are often associated with concentric resistance training, increases in the VL following eccentric resistance training have been reported^27,62^. Increases in fascicle angle can result in a greater physiological cross-sectional area and thus, greater force production capabilities^63^, which may contribute towards improvements in strength and power. Fascicle length did not increase in either training group, which is surprising as lengthening is also commonly reported following eccentric exercise^64^. However, a potential limitation of the present study was that a fixed probe was used to image muscle structure that was unable to capture full fascicle length. A trigonometric function was used to estimate fascicle length, however an increase in fascicle angle could result in a decreased fascicle length being calculated due to assuming that (1) fascicles are straight and (2) muscle thickness is consistent, with similar trends evidenced by Blazevich et al.^65^ who reported decreases in fascicle angle during detraining and consequently, increases in fascicle length. Another limitation to the study is that there was a slight sex imbalance between training groups, which could have affected the magnitude of adaptations as older males tend to report larger increases in absolute strength and muscle size^66^; given the sample size, sub-group analyses of sex was not possible. Finally, it should be noted that at baseline, G1X displayed lower maximal isometric strength than G2X, whilst G2X displayed smaller muscle thickness than G1X, which may have resulted in a greater capacity for each group to improve in each metric; however these findings were not statistically different and should not have influenced the findings, although further research is warranted to confirm this. Therefore, given that the present study used a fixed probe position only capable of imaging approximately 30% of the total VL fascicle length, extended field-of-view ultrasonography should be used where possible when imaging muscles with long and/or curved fascicles to avoid systematic error.

To conclude, the present findings confirm that for independently living older adults with no mobility impairments, one weekly multi-joint eccentric resistance training session lasting a total of 12 min per session over a 12-week period was sufficient to improve muscle size, strength, and power (i.e. criteria that are required to diagnose sarcopenia and factors closely associated with falls). Potentially of greater importance was that the improvements in the once-weekly training group were comparable to those obtained from twice-weekly training, particularly in muscular function adaptations, indicative of a more efficient training modality. Current resistance training exercise frequency recommendations for older adults in the United Kingdom are two sessions per week^14^, which have been shown to improve muscular size and function, particularly when lower-limb multi-joint exercises are performed^67^, however as the current recommended resistance training guidelines of two weekly sessions are not well adhered to by older adults, the current findings have important clinical implications for exercise prescription to help improve the poor adherence rates whilst retaining efficacy. Thus, eccentric resistance training may be pivotal in developing a minimal-dose approach to resistance training to combat neuromuscular decline in older adults whilst overcoming several participation barriers that currently exist for older adults (e.g. logistical, temporal, health-related, or fear of falling). Although the training was performed seated, the neuromuscular adaptations translated to improvements in the ability to perform everyday tasks, however for these adaptations to occur in functionally capable older individuals, the training programme must be adhered to for more than six weeks. Future work should investigate the efficacy of, and adherence to, eccentric training of a lower intensity or volume in clinical populations that demonstrate sarcopenia, frailty, and other age-related co-morbidities, whereby the training may feel easier than “hard” to perform, with particular emphasis on the adaptive profile, adherence, and a minimum effective maintenance dose following the initial training period.

## Data Availability

The dataset that support the findings of this study are openly available on PURE 10.24339/3373b688-e811-4847-9f38-ccf09a9c843a.

## Abbreviations

ANCOVA: Analysis of co-variance
ANOVA: Analysis of variance
CERT: Consensus on exercise reporting template
CON: Control group
*d*: Cohen’s *d*
G1X: Once-weekly training group
G2X: Twice-weekly training group
MVIC: Maximal voluntary isometric contraction
*r*: Non-parametric pairwise effect size
RPE: Rate of perceived exertion
RTD: Rate of torque development
STS: Sit-to-stand
VL: *vastus lateralis*
η_p_^2^: Analysis of variance effect size

## References

[1] Smith, P. *et al. Childcare and Early Years Survey of Parents 2010*. https://assets.publishing.service.gov.uk/media/5a7c6d4c40f0b626628abfa8/OSR_12_2012_Updated_Jun13.pdf (2012).

[2] Age UK. *Volunteering*. https://www.ageuk.org.uk/globalassets/age-uk/documents/policy-positions/active-communities/volunteering-policy-position---december-2022.pdf (2020).

[3] ONS. People aged 65 years and over in employment, UK: January to March 2022 to April to June 2022. https://www.ons.gov.uk/employmentandlabourmarket/peopleinwork/employmentandemployeetypes/articles/peopleaged65yearsandoverinemploymentuk/januarytomarch2022toapriltojune2022#:~:text=In%20April%20to%20June%202022%2C%20those%20aged%2065%20years%20and,in%20January%20to%20March%202022. (2022).

[4] Goodpaster B (2006). The loss of skeletal muscle strength, mass, and quality in older adults: The health, aging and body composition study. J. Gerontol.: Med. Sci..

[5] Skelton D, Greig C, Davies J (1994). Strength, power and related functional ability of healthy people aged 65–89 years. Age Ageing.

[6] Rubenstein LZ (2006). Falls in older people: Epidemiology, risk factors and strategies for prevention. Age Ageing.

[7] Manini T, Clark B (2012). Dynapenia and aging: An update. J. Gerontol. A Biol. Sci. Med. Sci..

[8] Reid K, Fielding R (2012). Skeletal muscle power: A critical determinant of physical functioning in older adults. Exerc. Sport Sci. Rev..

[9] Zanker J (2023). Mortality, falls and slow walking speed are predicted by different muscle strength and physical performance measures in women and men. Arch. Gerontol. Geriatr..

[10] He, W., Goodkind, D. & Kowal, P. *An Aging World: 2015*. https://www.census.gov/content/dam/Census/library/publications/2016/demo/p95-16-1.pdf (2016).

[11] Pinedo-Villanueva R (2019). Health care costs associated with muscle weakness: A UK population-based estimate. Calcif. Tissue Int..

[12] WHO. *Healthy Ageing and Functional Ability*. https://www.who.int/news-room/questions-and-answers/item/healthy-ageing-and-functional-ability (2020).

[13] Borde R, Hortobágyi T, Granacher U (2015). Dose-response relationships of resistance training in healthy old adults: A systematic review and meta-analysis. Sports Med..

[14] Department of Health & Social Care. *UK Chief Medical Officers’ Physical Activity Guidelines*. https://assets.publishing.service.gov.uk/media/5d839543ed915d52428dc134/uk-chief-medical-officers-physical-activity-guidelines.pdf (2019).

[15] Bennie J (2016). Pumping iron in Australia: Prevalence, trends and sociodemographic correlates of muscle strengthening activity participation from a national sample of 195,926 adults. PLoS ONE.

[16] Baert V, Gorus E, Mets T, Geerts C, Bautmans I (2011). Motivators and barriers for physical activity in the oldest old: A systematic review. Ageing Res. Rev..

[17] Burton E (2017). Why do seniors leave resistance training programs?. Clin. Interv. Aging.

[18] Skelton, D. *et al. UK Physical Activity Guidelines: Review and Recommendations for Older Adults (Aged 65+ Years)*. (2018).

[19] Paschalis V, Theodorou AA, Koutedakis Y (2011). A weekly bout of eccentric exercise is sufficient to induce health-promoting effects. Med. Sci. Sports Exerc..

[20] Douglas J, Pearson S, Ross A, McGuigan M (2017). Chronic adaptations to eccentric training: A systematic review. Sports Med..

[21] Roig M, O’Brien K, Kirk G, Murray R (2009). The effects of eccentric versus concentric resistance training on muscle strength and mass in healthy adults: A systematic review with meta-analysis. Br. J. Sports Med..

[22] Friedmann-Bette B (2010). Effects of strength training with eccentric overload on muscle adaptation in male athletes. Eur. J. Appl. Physiol..

[23] Kim D, Oh S, Lim J (2022). Applications of eccentric exercise to improve muscle and mobility function in older adults. Ann. Geriatr. Med. Res..

[24] Hoppeler H (2016). Moderate load eccentric exercise; A distinct novel training modality. Front. Physiol..

[25] Isner-Horobeti M (2013). Eccentric exercise training: Modalities, applications and perspectives. Sports Med..

[26] Reeves N, Maganaris C, Longo S, Narici M (2009). Differential adaptations to eccentric versus conventional resistance training in older humans. Exp. Physiol..

[27] Kay A, Blazevich A, Fraser M, Ashmore L, Hill M (2020). Isokinetic eccentric exercise substantially improves mobility, muscle strength and size, but not postural sway metrics in older adults, with limited regression observed following a detraining period. Eur. J. Appl. Physiol..

[28] LaStayo P, Ewy G, Pierotti D (2003). The positive effects of negative work: Increased muscle strength and decreased fall risk in a frail elderly population. J. Gerontol. A Biol. Sci. Med. Sci..

[29] Harper SA, Thompson BJ (2021). Potential benefits of a minimal dose eccentric resistance training paradigm to combat sarcopenia and age-related muscle and physical function deficits in older adults. Front. Physiol..

[30] Cvečka J (2023). Benefits of eccentric training with emphasis on demands of daily living activities and feasibility in older adults: A literature review. Int. J. Environ. Res. Public Health.

[31] Nuzzo JL, Pinto MD, Kirk BJC (2024). Resistance exercise minimal dose strategies for increasing muscle strength in the general population: an overview. Sports Med..

[32] Crane J, Thompson B, Harrell D, Bressel D, Heath E (2020). Comparison of high versus low eccentric-based resistance training frequencies on short-term muscle function adaptations. J. Strength Cond. Res..

[33] Schulz K, Altman D, Moher D (2010). CONSORT 2010 statement: Updated guidelines for reporting parallel group randomised trials. BMC Med..

[34] Sakugawa R (2019). Effects of resistance training, detraining, and retraining on strength and functional capacity in elderly. Aging Clin. Exp. Res..

[35] Slade S, Dionne C, Underwood M, Buchbinder R (2016). Consensus on exercise reporting template (CERT): Explanation and elaboration statement. Br. J. Sports Med..

[36] Borg G (1998). Borg’s Perceived Exertion and Pain Scales.

[37] Takai Y (2009). Sit-to-stand test to evaluate knee extensor muscle size and strength in the elderly: A novel approach. J. Physiol. Anthropol..

[38] Frey-Law L (2012). Knee and elbow 3D strength surfaces: Peak torque-angle-velocity relationships. J. Appl. Biomech..

[39] Tillin N, Pain M, Folland J (2013). Explosive force production during isometric squats correlates with athletic performance in rugby union players. J. Sports Sci..

[40] Haff G, Ruben R, Lider J, Twine C, Cormie P (2015). A comparison of methods for determining the rate of force development during isometric Midthigh clean pulls. J. Strength Cond. Res..

[41] Tillin N, Jimenez-Reyes P, Pain M, Folland J (2010). Neuromuscular performance of explosive power athletes versus untrained individuals. Med. Sci. Sports Exerc..

[42] Maffiuletti N (2016). Rate of force development: Physiological and methodological considerations. Eur. J. Appl. Physiol..

[43] Thompson B (2019). Influence of signal filtering and sample rate on isometric torque—Time parameters using a traditional isokinetic dynamometer. J. Biomech..

[44] Field A (2009). Discovering Statistics Using SPSS.

[45] Morris S, DeShon R (2002). Combining effect size estimates in meta-analysis with repeated measures and independent-groups designs. Psychol. Methods.

[46] Kirn D, Reid K, Hau C, Phillips E, Fielding R (2016). What is a clinically meaningful improvement in leg-extensor power for mobility-limited older adults?. J. Gerontol. A Biol. Sci. Med. Sci..

[47] Barbat-Artigas S, Rolland Y, Zamboni M, Aubertin-Leheudre M (2012). How to assess functional status: A new muscle quality index. J. Nutr. Health Aging.

[48] Ramirez-Velez R (2022). Sit to stand muscle power reference values and their association with adverse events in Colombian older adults. Sci. Rep..

[49] Loprinzi P (2016). Lower extremity muscular strength, sedentary behavior, and mortality. Age (Omaha).

[50] Spencer S, Thompson B, Bressel E, Louder T, Harrell D (2023). Transfer effects of a multiple-joint isokinetic eccentric resistance training intervention to nontraining-specific traditional muscle strength measures †. Sports.

[51] Lockhart T (2013). Biomechanics of Human Gait—Slip and Fall Analysis. Encyclopedia of Forensic Sciences.

[52] Robinovitch SN (2013). Video capture of the circumstances of falls in elderly people residing in long-term care: An observational study. The Lancet.

[53] Svanström L (1974). Falls on stairs: An epidemiological accident study. Scand. J. Soc. Med..

[54] Hody S, Croisier J, Bury T, Rogister B, Leprince P (2019). Eccentric muscle contractions: Risks and benefits. Front. Physiol..

[55] Hyldahl R, Hubal M (2014). Lengthening our perspective: Morphological, cellular, and molecular responses to eccentric exercise. Muscle Nerve.

[56] LaStayo P (2014). Eccentric exercise in rehabilitation: Safety, feasibility, and application. J. Appl. Physiol..

[57] Baxter B, Baross A, Ryan D, Wright B, Kay A (2023). The acute and repeated bout effects of multi-joint eccentric exercise on physical function and balance in older adults. Eur. J. Appl. Physiol..

[58] Clarkson P, Nosaka K, Braun B (1992). Muscle function after exercise-induced muscle damage and rapid adaptation. Med. Sci. Sports Exerc..

[59] Burt D, Lamb K, Nicholas C, Twist C (2013). Effects of repeated bouts of squatting exercise on sub-maximal endurance running performance. Eur. J. Appl. Physiol..

[60] Nuzzo J, Pinto M, Nosaka K, Steele J (2022). How much stronger are muscles eccentrically than concentrically? Meta-analysis of the influences of sex, age, joint action, and velocity. SportRxiv.

[61] Morton RW, Colenso-Semple L, Phillips S (2019). Training for strength and hypertrophy: An evidence-based approach. Curr. Opin. Physiol..

[62] Benford J, Hughes J, Waldron M, Theis N (2021). Concentric versus eccentric training: Effect on muscle strength, regional morphology, and architecture. Transl. Sports Med..

[63] Folland J, Williams A (2007). The adaptations to strength training morphological and neurological contributions to increased strength. Sports Med..

[64] Franchi MV, Reeves ND, Narici MV (2017). Skeletal muscle remodeling in response to eccentric vs. concentric loading: Morphological, molecular, and metabolic adaptations. Front. Physiol..

[65] Blazevich A, Cannavan D, Coleman D, Horne S (2007). Influence of concentric and eccentric resistance training on architectural adaptation in human quadriceps muscles. J. Appl. Physiol..

[66] Jones M, Wewege M, Hackett D, Keogh J, Hagstrom A (2021). Sex differences in adaptations in muscle strength and size following resistance training in older adults: A systematic review and meta-analysis. Sports Med..

[67] Fragala MS (2019). Resistance training for older adults: Position statement from the national strength and conditioning association. J. Strength Cond. Res..

